# Comparison of adaptive versions of the Hong Kong-specific criteria and 2015 Beers criteria for assessing potentially inappropriate medication use in Hong Kong older patients

**DOI:** 10.1186/s12877-021-02324-5

**Published:** 2021-06-21

**Authors:** Huanyu ZHANG, Eliza L. Y. WONG, Samuel Y. S. WONG, Patsy Y. K. CHAU, Benjamin H. K. YIP, Roger Y. N. CHUNG, Eric K. P. LEE, Francisco T. T. LAI, Eng-kiong YEOH

**Affiliations:** The Jockey Club School of Public Health and Primary Care, Faculty of Medicine, The Chinese University of Hong Kong, Shatin, New Territories, Hong Kong

**Keywords:** Potentially inappropriate medication, Hong Kong-specific criteria, Beers criteria, Older adults, Hong Kong

## Abstract

**Background:**

The Hong Kong-specific criteria have been established in 2019 to assess potentially inappropriate medication (PIM) use in older adults and improve the local prescribing quality. The aim of this study was to compare the adaptive versions of the Hong Kong-specific criteria and 2015 Beers criteria for assessing the prevalence and correlates of PIM use in Hong Kong older patients.

**Methods:**

A cross-sectional study was performed from January 1, 2014 to December 31, 2014 using the Hospital Authority (HA) database. A total of 489,301 older patients aged 65 years and older visiting general outpatient clinics (GOPCs) during the study period were included in the study. Two categories of PIM use included in the Hong Kong-specific criteria and 2015 Beers criteria, i.e. PIMs independent of diagnoses and PIMs considering specific medical conditions, were adapted to assess the prevalence of PIM use among the study sample. Characteristics of PIM users and the most frequently prescribed PIMs were investigated for each set of the criteria. Factors associated with PIM use were identified using the stepwise multivariable logistic regression analysis.

**Results:**

The adaptive Hong Kong-specific criteria could detect a higher prevalence of patients exposed to at least one PIM than that assessed by the adaptive Beers criteria (49.5% vs 47.5%). Meanwhile, the adaptive Hong Kong-specific criteria could identify a higher rate of patients exposed to PIMs independent of diagnoses (48.1% vs 46.8%) and PIMs considering specific medical conditions (7.3% vs 4.9%) compared with that of the adaptive Beers criteria. The most frequently prescribed PIMs detected by the adaptive Beers criteria were all included in the adaptive Hong Kong-specific criteria. The strongest factor associated with PIM use was number of different medications prescribed. Patients with female gender, aged 65 ~ 74 years, a larger number of GOPC visits, and more than six diagnoses were associated with greater risk of PIM use, whereas advancing age was associated with lower risk of PIM use.

**Conclusions:**

The adaptive Hong Kong-specific criteria could detect a higher prevalence of PIM use than the adaptive Beers criteria in older adults visiting GOPCs in Hong Kong. It is necessary to update the prevalence and correlates of PIM use regularly in older adults to monitor the burden of PIM use and identify vulnerable patients who need further interventions.

**Supplementary Information:**

The online version contains supplementary material available at 10.1186/s12877-021-02324-5.

## Background

Potentially inappropriate medication (PIM) use occurs when patients are prescribed a drug that has a high risk of adverse drug events (ADEs) as compared to its potential benefits, especially when safer or more effective therapeutic alternatives are available [1]. These ADEs could increase the risk of mortality, morbidity, hospitalization and thus increase healthcare expenditures [2, 3]. The aging population is particularly at risk for PIM use because the physiologic changes in pharmacokinetics and pharmacodynamics in older adults can affect their body’s capacity to handle medications [4]. In addition, many older adults suffer from comorbidity, which often requires the prescription of multiple medications [4, 5]. Several studies indicated that the use of multiple medications was associated with greater risk of PIM use in older adults [6, 7]. Since the prescribing of inappropriate medications is an important cause of preventable ADEs [8], it is necessary to facilitate the deprescribing of inappropriate medications and optimize the prescription of drug therapies to reduce the emergence of ADEs and improve the appropriateness of prescribing.

In Hong Kong, the Hospital Authority (HA) is responsible for the management of all the public health services, including public hospitals, Specialist Out-patient Clinics (SOPCs), and General Out-patient Clinics (GOPCs) [9]. Approximately 30% of outpatient consultations are provided by public sectors in Hong Kong [10]. With no system for patients to register with a particular general practitioner (GP), patients can consult different GPs from the public and private sectors in Hong Kong [11]. Additionally, older patients with comorbidity may consult multiple specialty or sub-specialty clinics [12]. Poor communication between doctors practicing in different settings or even between specialties practicing in the same setting can give rise to inappropriate prescribing [13]. In the public sector, the average time of GOPC consultations lasts three to five min, and such a short time makes it difficult to provide adequate counseling and medication review [14]. Despite the fragmented care and short consultation time, the lack of separation of prescribing and dispensing in Hong Kong has muted the role of pharmacists [15]; patients are at greater risk of PIM use without health professionals overseeing their medication lists. Therefore, it is crucial to quantify the burden of PIM use in public outpatient settings among older adults in Hong Kong and identify vulnerable patients for further interventions.

Explicit PIM lists are defined as drug or disease oriented firm standards developed from literature review, expert opinions or consensus techniques [16]. They can be applied to large samples of people to assess the burden of PIM use from a population level [17]. The Beers criteria were the first set of explicit criteria established in the United States [1] to assess PIM use in older adults and updated on a routine basis since 2012 [18, 19]. A large number of studies have employed the Beers criteria to assess the prevalence of PIM use in older adults in different countries beyond the US [20–23]. Because different countries may vary in their approved drugs, clinical practice and health system regulations, several country-specific PIM lists have been developed to improve the local prescribing quality [24, 25]. The Hong Kong-specific criteria [26] were established based on nine sets of criteria published across North America, Europe and Asia, one of which were the 2015 updated Beers criteria [18]. Although the Hong Kong-specific PIM list has been validated by a two-round modified Delphi process, it has not been applied to assess the prevalence and correlates of PIM use among older adults in Hong Kong. Previous studies conducted in Hong Kong have used explicit tools to assess the extent of PIM use in older adults [27, 28]. However, these studies have merely employed limited samples; fewer studies have been performed in a general, territory-wide population. Hence, the main objective of this study is to compare the ability of the Hong Kong-specific criteria to detect PIMs against the Beers criteria in older adults visiting GOPCs in Hong Kong from a population level. The secondary objective is to investigate the factors associated with PIM use and the most frequently prescribed PIMs identified by the two sets of criteria.

## Methods

### Data source

A cross-sectional study was conducted from January 1, 2014 to December 31, 2014 using the HA database. Patients’ electronic health records were collected by doctors using the HA computer system. Since the HA computer system includes all the patients who use public health services in Hong Kong, the data extracted from the HA database are highly reliable. Each patient was allocated a unique identity code so as to link up their clinical records across different datasets.

In this study, a cohort of older adults aged 65 years or older visiting GOPCs in 2014 were extracted from the general outpatient dataset. All the diagnoses for each patient visiting GOPCs during the study period were considered in the current study and coded using the International Classification of Primary Care, Second edition (ICPC-2) system. The prescribing information of the study population was collected from the medication datasets, which included drug prescriptions in the public primary care setting. The medication datasets provided information on generic drug names of medications originating from the HA drug formulary without any detail of indication, dose or therapy duration. The records on healthcare service use including GOPC visits and hospital admissions were extracted from the general outpatient dataset and inpatient dataset, respectively. Each consultation episode per patient during the study period was identified by a unique sequence number. The data we used for this study were retrospective, pre-existing and de-identified; any sensitive information on patients, doctors or clinics was not accessible.

### Identification of PIM use

PIM use in the current study was identified by the 2015 Beers criteria and Hong Kong-specific criteria, respectively. The operational definition of PIM use for this study used two categories of PIMs, namely, PIMs independent of diagnoses and PIMs considering specific medical conditions, for they were both included in the two sets of criteria. Since the prescription records in the database were originated from the HA drug formulary, the availability of PIMs in the context of the public primary care setting was examined by this formulary. PIMs that were not covered by the HA drug formulary were excluded from the current study. Due to the incomplete prescribing information provided by the HA database, PIMs with any restriction or exception in terms of dose, indication or therapy duration defined by the two sets of criteria were not taken into account. Additionally, the certain PIMs considering specific medical conditions without an exclusive ICPC-2 code corresponding to its medical condition were also excluded from the current study. After applying the inclusion and exclusion criteria, the adaptive versions of the Beers criteria and Hong Kong-specific criteria were presented in the Additional file 1 and Additional file 2, separately. The identification of PIM use was conducted by computer according to the two adaptive PIM lists. Patients with at least one PIM use during the study period were defined as PIM users.

### Statistical analysis

The prevalence and associated factors of PIM use were analyzed separately for the two sets of criteria. The most common PIMs that were prescribed in more than 1% of the study population were listed for each set of criteria. The following patient variables were investigated for their association with PIM use: gender, age, number of different drugs prescribed, number of diagnoses, number of GOPC visits within the year, and number of hospital admissions within the year. Chi-square tests were performed for categorical variables to test the correlations between patient characteristics and PIM use. Stepwise multivariable logistic regression models were used to identify factors associated with PIM use at GOPCs in older adults. Adjusted odds ratios (AORs) with 95% confidence intervals (CIs) were reported. The statistical significance was set at *p* value < 0.05. R version 3.6.3 software was used for all statistical analyses.

## Results

As shown in Table 1, the study population included a total of 489,301 older adults visiting GOPCs in 2014, which accounted for nearly half of the total aging population in Hong Kong. The mean age of the patients was 75.1 ± 7.7 years (27.9% aged ≥80), and 45.6% were males. Nearly 40% of the patients were prescribed 10 and more different drugs and more than half (59.3%) had more than 3 diagnoses. The mean number of GOPC visits for each patient was 4.1 ± 2.5, and 26.8% of the patients had at least one hospital admission within the year.

**Table 1:** Characteristics of the older patients visiting GOPCs in 2014

Characteristics	N (%) or mean (SD)
**All**	**489,301**
**Gender**	
Male	223,142 (45.6%)
Female	266,159 (54.4%)
**Age**	75.1 (7.7)
65 ~ 69	159,424 (32.6%)
70 ~ 74	98,683 (20.2%)
75 ~ 79	94,707 (19.4%)
80 ~ 84	74,732 (15.3%)
85+	61,755 (12.6%)
**No. of drugs**	9.6 (7.0)
0 ~ 3	72,403 (14.8%)
4 ~ 6	118,136 (24.1%)
7 ~ 9	106,737 (21.8%)
10 ~ 12	72,224 (14.8%)
> 12	119,801 (24.5%)
**No. of diagnoses**	4.3 (2.1)
1	7803 (1.6%)
2	87,941 (18.0%)
3	103,060 (21.1%)
4 ~ 6	229,071 (46.8%)
> 6	61,426 (12.6%)
**No. of GOPC visits within the year**	4.1 (2.5)
1	67,499 (13.8%)
2 ~ 3	128,476 (26.3%)
4 ~ 5	196,686 (40.2%)
6+	96,640 (19.8%)
**No. of hospital admissions within the year**	0.6 (1.8)
0	358,489 (73.3%)
1	68,434 (14.0%)
2+	62,378 (12.8%)

The basic characteristics and the ability to detect PIMs between the Beers criteria and the Hong Kong-specific criteria were compared in Table 2. Overall, the total number of addressed PIM statements between the Beers criteria and the Hong Kong-specific criteria are identical (163 vs 163). However, after the adaption to the context of the HA database, only 43% of the addressed statements from the Beers criteria were included in the current study, while 61% of the statements from the Hong Kong-specific criteria were considered. In summary, the adaptive Hong Kong-specific criteria could detect a higher prevalence of PIM use than that assessed by the adaptive Beers criteria (49.5% vs 47.5%) in older adults visiting GOPCs in Hong Kong. For each category of PIM use addressed in this study, the adaptive Hong Kong-specific criteria could identify a higher rate of patients exposed to PIMs independent of diagnoses (48.1% vs 46.8%) and PIMs considering specific medical conditions (7.3% vs 4.9%) compared with that evaluated by the adaptive Beers criteria.

**Table 2:** Comparison of basic characteristics and the ability to detect PIMs between the Beers criteria vs. Hong Kong-specific criteria

Characteristics	Beers criteria	Hong Kong-specific criteria
Year	2015	2019
Country	USA	Hong Kong, China
No. of PIMs independent of diagnoses	113	76
No. (%) of adaptive PIMs independent of diagnoses	26 (23.0%)	34 (44.7%)
No. of PIMs considering specific conditions	50	87
No. (%) of adaptive PIMs considering specific conditions	44 (88.0%)	66 (75.9%)
No. (%) of patients exposed to adaptive PIMs independent of diagnoses	228,966 (46.8%)	235,397 (48.1%)
No. (%) of patients exposed to adaptive PIMs considering specific medical conditions	24,144 (4.9%)	35,568 (7.3%)
Total no. (%) of patients exposed to at least one PIM	232,445 (47.5%)	241,982 (49.5%)

The characteristics of PIM users assessed by the adaptive versions of the Beers criteria and Hong Kong-specific criteria were described in Table 3. Among the 232,445 (47.5%) PIM users assessed by the adaptive Beers criteria, 62.9% were prescribed one PIM, followed by 27.5% with two PIMs, 7.5% with three PIMs, and 2.1% with more than three PIMs prescribed. On the other hand, among the 241,982 (49.5%) PIM users assessed by the adaptive Hong Kong-specific criteria, 58.7% were prescribed one PIM, followed by 28.2% with two PIMs, 9.4% with three PIMs, and 3.7% with more than three PIMs prescribed. Among the PIM users assessed by the adaptive Beers criteria, those aged 65 ~ 69 had the highest risk of receiving at least one PIM. By contrast, patients aged 85 years or older were at the highest risk of being prescribed PIMs listed in the adaptive Hong Kong-specific criteria. Patients having a larger number of health care service utilization including GOPC visits and hospital admissions within the year had a greater risk of receiving PIMs. According to the two sets of criteria, the proportions of patients receiving PIMs increased to over 70% when patients had more than 6 diagnoses and 12 medications prescribed.

**Table 3:** Description of PIM users assessed by the adaptive Beers criteria vs. adaptive Hong Kong-specific criteria

Variables	Adaptive Beers criteria	***p*** value	Adaptive Hong Kong-specific criteria	***p*** value
**No. of PIM users**	232,445 (47.5%)		241,982 (49.5%)	
**No. of concurrent PIMs**		< 0.001		< 0.001
1	146,240 (62.9%)		141,969 (58.7%)	
2	64,025 (27.5%)		68,145 (28.2%)	
3	17,377 (7.5%)		22,818 (9.4%)	
> 3	4804 (2.1%)		9050 (3.7%)	
**Gender**		< 0.001		< 0.001
Male	101,764 (45.6%)		105,616 (47.3%)	
Female	130,681 (49.1%)		136,366 (51.2%)	
**Age**		0.076		< 0.001
65 ~ 69	76,252 (47.8%)		77,383 (48.5%)	
70 ~ 74	46,552 (47.2%)		47,908 (48.6%)	
75 ~ 79	44,862 (47.4%)		46,941 (49.6%)	
80 ~ 84	35,652 (47.7%)		37,903 (50.7%)	
85+	29,127 (47.2%)		31,847 (51.6%)	
**No. of drugs**		< 0.001		< 0.001
0 ~ 3	7423 (10.3%)		7588 (10.5%)	
4 ~ 6	37,099 (31.4%)		37,845 (32.0%)	
7 ~ 9	51,842 (48.6%)		53,324 (50.0%)	
10 ~ 12	45,314 (62.7%)		46,787 (64.8%)	
> 12	90,767 (75.8%)		96,438 (80.5%)	
**No. of diagnoses**		< 0.001		< 0.001
1	3720 (47.7%)		3883 (49.8%)	
2	32,890 (37.4%)		34,743 (39.5%)	
3	41,064 (39.8%)		42,766 (41.5%)	
4 ~ 6	109,321 (47.7%)		113,659 (49.6%)	
> 6	45,450 (74.0%)		46,931 (76.4%)	
**No. of GOPC visits within the year**		< 0.001		< 0.001
1	25,403 (37.6%)		27,561 (40.8%)	
2 ~ 3	53,661 (41.8%)		56,273 (43.8%)	
4 ~ 5	87,427 (44.5%)		90,731 (46.1%)	
6+	65,954 (68.3%)		67,417 (69.8%)	
**No. of hospital admissions within the year**		< 0.001		< 0.001
0	159,721 (44.6%)		162,103 (45.2%)	
1	35,718 (52.2%)		38,127 (55.7%)	
2+	37,006 (59.3%)		41,752 (66.9%)	

The factors associated with having at least one PIM use were identified in the multivariable logistic regression analysis by applying the adaptive versions of the Beers criteria and Hong Kong-specific criteria, respectively (Table 4). Females were at greater risk of receiving PIMs than males. The risk of being prescribed PIMs decreased with age; patients aged 65 ~ 69 were at the highest risk of receiving PIMs. The strongest factor associated with PIM use was number of different drugs prescribed, especially when using the adaptive Hong Kong-specific criteria (AOR = 34.96, 95% CI = 33.96 ~ 36.01 for > 12 drugs vs. 3 or less). A larger number of GOPC visits within the year was associated with a higher likelihood of PIM use. Patients with more than six diagnoses were at greater risk of receiving PIMs compared with those had only one diagnosis.

**Table 4:** Factors associated with having at least one PIM use based on multivariable logistic regression analysis

Variables	AOR[95%CI]	
	Adaptive Beers criteria	Adaptive Hong Kong-specific criteria
**Gender (M:F)**	0.83 [0.82 ~ 0.84]***	0.82 [0.81 ~ 0.83]***
**Age (Ref: 65 ~ 69)**		
70 ~ 74	0.79 [0.78 ~ 0.80]***	0.81 [0.80 ~ 0.83]***
75 ~ 79	0.69 [0.68 ~ 0.70]***	0.73 [0.72 ~ 0.74]***
80 ~ 84	0.61 [0.60 ~ 0.63]***	0.66 [0.65 ~ 0.68]***
85+	0.51 [0.50 ~ 0.52]***	0.58 [0.56 ~ 0.59]***
**No. of drugs (Ref:** 3 **or less)**		
4 ~ 6	4.16 [4.04 ~ 4.27]***	4.20 [4.08 ~ 4.31]***
7 ~ 9	8.60 [8.37 ~ 8.85]***	8.90 [8.66 ~ 9.15]***
10 ~ 12	14.86 [14.43 ~ 15.31]***	15.82 [15.36 ~ 16.30]***
> 12	27.48 [26.70 ~ 28.29]***	34.96 [33.96 ~ 36.01]***
**No. of diagnoses (Ref:** 1)		
2	1.12 [1.06 ~ 1.19]***	1.14 [1.08 ~ 1.21]***
3	1.00 [0.94 ~ 1.05]	1.02 [0.97 ~ 1.08]
4 ~ 6	0.90 [0.86 ~ 0.95]***	0.93 [0.88 ~ 0.98]**
> 6	1.37 [1.29 ~ 1.44]***	1.45 [1.37 ~ 1.53]***
**No. of GOPC visits within the year (Ref:** 1)		
2 ~ 3	1.18 [1.15 ~ 1.20]***	1.10 [1.07 ~ 1.13]***
4 ~ 5	1.20 [1.17 ~ 1.22]***	1.09 [1.06 ~ 1.12]***
6+	1.84 [1.79 ~ 1.90]***	1.61 [1.56 ~ 1.65]***

*p < 0.05

**p < 0.01

***p < 0.001

Table 5 presented thirteen PIM statements that were prescribed in over 1% of the study sample. All these PIM statements could be identified by the adaptive Hong Kong-specific criteria, while ten of these PIM statements were identified by the adaptive Beers criteria. According to the findings assessed by the two sets of criteria, the most frequently prescribed PIMs independent of diagnoses included chlorpheniramine (35.41%), promethazine (8.73%), diphenhydramine (8.44%), and methyldopa (4.07%), while the most common PIMs considering specific medical conditions were the medications exacerbating benign prostatic hyperplasia (2.60%), and medications worsening lower urinary tract symptoms (1.26%). The most frequently prescribed PIMs that were unique to the adaptive Hong Kong-specific criteria included metoclopramide (3.01%), zopiclone (2.15%), and medications exacerbating chronic constipation (2.50%).

**Table 5:** The most frequently prescribed PIMs in older patients visiting GOPCs in 2014

PIMs independent of diagnoses			
**PIMs**	**Prevalence**	**Adaptive Beers criteria**	**Adaptive Hong Kong-specific criteria**
Chlorpheniramine	35.41%	**✓**	**✓**
Promethazine	8.73%	**✓**	**✓**
Diphenhydramine	8.44%	**✓**	**✓**
Methyldopa	4.07%	**✓**	**✓**
Metoclopramide	3.01%		**✓**
Zopiclone	2.15%		**✓**
Dexchlorpheniramine	1.92%	**✓**	**✓**
Lorazepam	1.16%	**✓**	**✓**
Indomethacin	1.14%	**✓**	**✓**
Amitriptyline	1.07%	**✓**	**✓**
**PIMs due to disease-drug interactions**			
**PIMs**	**Prevalence**	**Adaptive Beers criteria**	**Adaptive Hong Kong-specific criteria**
Benign prostatic hyperplasia-anticholinergics, TCAs	2.60%	**✓**	**✓**
Chronic constipation-anticholinergics, CCBs, methyldopa, opioids, TCAs	2.50%		**✓**
Lower urinary tract symptoms-anticholinergics, chlorpromazine, TCAs	1.26%	**✓**	**✓**

## Discussion

This is the first population-based study assessing PIM use in older adults using an explicit tool developed in Hong Kong. The ability of the Hong Kong-specific criteria in detecting PIMs was also compared with another set of well-established criteria, i.e. the Beers criteria. Both adaptive versions of the criteria identified a high prevalence of PIM use in older adults vising GOPCs in Hong Kong. Furthermore, the adaptive Hong Kong-specific criteria could detect a higher prevalence of PIM use, including PIMs independent of diagnoses and PIMs considering specific medical conditions, than that evaluated by the adaptive Beers criteria in the local context. Similar patterns of risk factors associated with PIM use were identified by applying the two sets of criteria. In the multivariable regression analysis, the findings showed that PIM use was associated with female gender, larger number of drugs prescribed, more frequent GOPC visits, and more than six diagnoses, which were generally consistent with the results from previous studies [29–31]. However, most of the previous studies suggested that PIM use was associated with advancing age [29], whereas the current study showed that the risk of PIM use decreased with age. A recent study conducted in the US indicated that the risk of receiving a PIM decreased with age in older adults [31]. Nonetheless, it is difficult to make a proper comparison between different studies because of the difference in terms of study samples, assessment tools, drug availability, and clinical settings.

The prevalence rates of PIM use assessed in the current study were relatively higher than those reported in western countries in the primary care setting (ranging from 2.90 to 40.7%) [20, 29] as well as the prevalence estimates (ranging from 30.3 to 38.6%) [26, 27] previously reported in Hong Kong. The high rates of PIM use in older adults visiting GOPCs in Hong Kong could be explained by several reasons. First, polypharmacy, generally defined as five or more drugs prescribed or dispensed, could increase the risk of PIM use in older adults. In Hong Kong, more than half of the aging population visiting GOPCs was exposed to polypharmacy based on the findings of this study. Second, the HA provided unrestricted access to GOPC services, including drug prescriptions, which are highly subsidized by the government. Consequently, older adults made more frequent use of public services, which could elevate the risk of PIM use. Third, prescribers may lack of awareness of the risk of PIM use for older adults. For example, the prescription of first-generation antihistamines is common in Hong Kong because the general practitioners at GOPCs are used to prescribing this medication for the treatment of colds. However, prescribers should be more cautious when prescribing first-antihistamines to older adults because the risk may outweigh its benefits.

Among the most frequently prescribed PIMs in older adults visiting GOPCs, all the PIMs identified by the adaptive Beers criteria were included in the adaptive Hong Kong-specific criteria. Despite the similarities on the most common PIMs assessed by the two sets of criteria, the adaptive Hong Kong-specific criteria could detect certain PIMs that the adaptive Beers criteria failed to identify, involving metoclopramide, zopiclone, and medications exacerbating chronic constipation. In fact, metoclopramide was incorporated in the 2015 Beers criteria, however, it was excluded from the adaptive Beers criteria for this drug could be used exceptionally for the treatment of gastroparesis in older adults according to the consensus of the Beers expert panel. In addition, the criterion of medications exacerbating chronic constipation has been removed from the Beers criteria since 2012 because the Beers expert panel believes this drug-disease combination is not particularly problematic to older adults. Furthermore, the adaptive Beers criteria failed to detect zopiclone in the context of Hong Kong mainly because this drug was not available in the drug market of the US. Although the Hong Kong-specific criteria were established in the context of HA drug formulary, most of the individual PIMs in this PIM list were also included in the Beers criteria and other country-specific PIM criteria. Therefore, it is feasible to apply the Hong Kong-specific criteria to measure the appropriateness of prescribing in other regions and make international comparisons.

The study population of this research was representative by including nearly half of older adults in Hong Kong. Few studies have used population-based data to assess the burden of PIM use in Hong Kong. Furthermore, most of previous studies using explicit criteria for PIM evaluations only employed the category of PIMs independent of diagnoses due to incomplete clinical information [29]. In addition to generally avoided PIMs, this study also identified PIMs considering specific medical conditions in older adults, which provided a more comprehensive view of PIM use in Hong Kong. However, this study also has some limitations. First, due to the exclusion of PIMs with any restriction or exception in terms of dose, indication or therapy duration, the prevalence rates of PIM use in the current study was conservative. Although all the PIMs included in the Hong Kong-specific criteria were available in the HA drug formulary, some of the medical conditions in the category of PIMs considering specific medical conditions cannot be identified in the primary care setting with the ICPC-2 coding system. Second, the majority of outpatient consultations were provided by private clinics in Hong Kong, thus older patients may have other sources of PIM use in the primary care setting without evaluations at the population level. Third, the Beers criteria were updated in 2019 with addition, removal, and modification of the PIM list [19]. Meanwhile, the HA drug formulary was updated quarterly during each year by including more cost-effective drugs in the formulary. These changes could influence the measurement of PIM use in older adults in Hong Kong, yet have not been considered in the current study. Given the changes in the availability of medications and the release of new evidence defining PIMs, it is necessary to update the prevalence rates of PIM use in older adults on a routine basis. Fourth, the relationship between explicit PIM criteria and adverse outcomes should be externally validated so as to explore the negative effects of PIM use on patients’ health. The Beers criteria have been proved to be associated with multiple patient-related outcomes such as adverse drug reactions [32], hospitalization, falls, and mortality [3]. Future studies need to focus on validating the association between the Hong Kong-specific criteria and patient-related health outcomes in both clinical and research settings.

In this study, the key findings can inform policies for the healthcare system of Hong Kong and contribute to the improvement of the quality of prescribing in the local context. Analysis of the prevalence and correlates of PIM use in the current study can contribute to the enhancement of prescribing quality by quantifying the burden of PIM use in Hong Kong at the population level and identifying vulnerable patients who require further interventions. Education on prescribers should be strengthened to raise their awareness on the importance of pharmacotherapy for older patients, particularly in older adults who are exposed to polypharmacy. Apart from preparing and distributing medications, the role of pharmacists could be expanded to review medication lists and provide counseling services for vulnerable older patients in hospital and community settings. Because all the medications from the Hong Kong-specific criteria are available in the HA drug formulary, this assessment tool can be integrated into the HA computer system for warning the use of high-risk medications and providing therapeutic alternatives. The regular update of the HA drug formulary should consider including fewer PIMs listed in the Hong Kong-specific criteria and purchasing more alternatives.

## Conclusions

In conclusion, the adaptive Hong Kong-specific criteria could detect a higher prevalence of PIM use than the adaptive Beers criteria in older adults visiting GOPCs in Hong Kong. Similar patterns of risk factors associated with PIM use were identified by applying the two sets of criteria. Patients with female gender, polypharmacy, larger number of GOPC visits, and more than six diagnoses were more likely to receive PIMs, whereas advancing age was associated with lower risk of PIM use. It is necessary to update the prevalence and correlates of PIM use on a routine basis in Hong Kong older patients to monitor the burden of PIM use and identify vulnerable patients who need further interventions.

## Supplementary Information

**Additional file 1.** Adaptive Beers PIM list

**Additional file 2.** Adaptive Hong Kong-specific PIM list

## Data Availability

All data generated or analyzed during this study are included in this published article and its’ supplementary information files.

## Abbreviations

PIM: Potentially inappropriate medication
ADE: Adverse drug event
HA: Hospital authority
GOPC: General outpatient clinics
GP: General practitioner
ICPC-2: International classification of primary care, second edition
