# Supplementary material for: Comparison of adaptive versions of the Hong Kong-specific criteria and 2015 Beers criteria for assessing potentially inappropriate medication use in Hong Kong older patients
Source: BMC Geriatr. 2021 Jun 21;21:379. doi: 10.1186/s12877-021-02324-5 (PMC8218399; doi:10.1186/s12877-021-02324-5)
Supplement: Supplementary file 1 — Additional file 1. Adaptive Beers PIM list [file 12877_2021_2324_MOESM1_ESM.docx]

**Additional file 1. Adaptive Beers criteria for assessing PIM use in older adults visiting GOPCs in Hong Kong**

| **PIMs independent of diagnoses** | | |
| --- | --- | --- |
| **Organ system** | **Medication class** | **Medication** |
| Anticholinergics | First-generation antihistamines | chlorpheniramine, cyproheptadine,  dexchlorpheniramine, dimenhydrinate  hydroxyzine, promethazine, diphenhydramine |
| Cardiovascular | Central alpha blockers | methyldopa |
|  | Nifedipine | nifedipine (immediate release) |
| Central nervous system | Antidepressants | amitriptyline, clomipramine, imipramine  nortriptyline, paroxetine, trimipramine |
|  | Benzodiazepines (*Short- and intermediate- acting*) | alprazolam, lorazepam, triazolam |
|  | Benzodiazepines (*Long- acting*) | chlordiazepoxide, clonazepam, diazepam |
|  | Nonbenzodiazepine | zolpidem |
|  | Megestrol | megestrol |
| Pain medication | NSAIDs | indomethacin |
|  | Ketorolac | ketorolac, includes parenteral |
|  | Skeletal muscle relaxants | orphenadrine |
| **PIMs due to disease-drug interactions** | | |
| **Disease or syndrome** | **ICPC-2 code** | **Medication** |
| Heart failure | K77 | NSAIDs and COX-2 inhibitors, thiazolidinediones (pioglitazone, rosiglitazone), cilostazol |
| Syncope | A06 | AChEIs, peripheral alpha-1 blockers (doxazosin, prazosin, terazosin), tertiary TCAs, chlorpromazine, thioridazine, olanzapine |
| Chronic seizures or epilepsy | N07; N88 | bupropion, chlorpromazine, clozapine, olanzapine, thioridazine, tramadol |
| Delirium | P71 | anticholinergics, antipsychotics, benzodiazepines, chlorpromazine, corticosteroids, H2-receptor antagonists (famotidine, ranitidine), sedative hypnotics |
| Dementia or cognitive impairment | P70 | anticholinergics, benzodiazepines, H2-receptor antagonists (famotidine, ranitidine), zolpidem, antipsychotics |
| History of falls or fractures | L72~L76 | anticonvulsants, antipsychotics, benzodiazepines, zolpidem, TCAs, SSRIs, opioids |
| Insomnia | P06 | oral decongestants (pseudoephedrine, phenylephrine), stimulants (methylphenidate), theobromines (theophylline, caffeine) |
| Parkinson disease | N87 | antipsychotics (except aripiprazole, quetiapine, clozapine) antiemetics (metoclopramide, prochlorperazine, promethazine) |
| History of gastric or duodenal ulcers | D85; D86 | Non-COX-2 selective NSAIDs |
| Chronic kidney disease | U99 | NSAIDs |
| Urinary incontinence all types | U04 | oestrogen, peripheral alpha-1 blockers (doxazosin, prazosin, terazosin) |
| Lower urinary tract symptoms, benign prostatic hyperplasia | U02; U05; U07; U13; U29; Y06; Y85 | anticholinergics |
