# Supplementary material for: Comparison of adaptive versions of the Hong Kong-specific criteria and 2015 Beers criteria for assessing potentially inappropriate medication use in Hong Kong older patients
Source: BMC Geriatr. 2021 Jun 21;21:379. doi: 10.1186/s12877-021-02324-5 (PMC8218399; doi:10.1186/s12877-021-02324-5)
Supplement: Supplementary file 2 — Additional file 2. Adaptive Hong Kong-specific PIM list [file 12877_2021_2324_MOESM2_ESM.docx]

**Additional file 2. Adaptive Hong Kong-specific criteria for assessing PIM use in older adults visiting GOPCs in Hong Kong**

| **PIMs independent of diagnoses** | | |
| --- | --- | --- |
| **ATC code** | **Medication class** | **Medications** |
| **A03** | **Antispasmodics** | Propantheline, Metoclopramide |
| **C01** | **Antiarrhythmics** | Disopyramide |
| **C02** | **Antihypertensives** | Methyldopa |
| **C08** | **Calcium channel blockers** | Nifedipine |
| **C02; G04** | **Urologicals** | Oxybutynin |
| **M01** | **Antiinflammatory and antirheumatic products** | Indometacin, Ketorolac |
| **M03** | **Muscle relaxants** | Orphenadrine, Tolperisone |
| **N03BA; N05CD** | **Benzodiazepines** | Clonazepam, Diazepam, Chlordiazepoxide  Lorazepam, Bromazepam, Clobazam  Alprazolam, Nitrazepam, Flunitrazepam  Triazolam, Midazolam |
| **N05BB** | **Anxiolytics** | Hydroxyzine |
| **N05CF** | **Hypnotics and sedatives** | Zopiclone, Zolpidem |
| **N06AA** | **Antidepressants** | Imipramine, Clomipramine, Trimipramine  Amitriptyline, Nortriptyline |
| **R06** | **Antihistamines** | Chlorpheniramine, Cyproheptadine  Dexchlorpheniramine, Diphenhydramine  Promethazine |
| **PIMs considering specific medical conditions** | | |
| **ICPC-2** | **Medical conditions** | **Medication classes/ Medications** |
| **B83** | **Blood clotting disorders** | Aspirin, NSAIDs |
| **A06** | **Syncope** | Alpha-adrenoreceptor antagonists  Chlorpromazine, Methyldopa  TCAs, Thioridazine |
| **K77** | **Heart failure** | NSAIDs, Pioglitazone |
| **K80** | **Cardiac arrhythmia** | Antipsychotics |
| **P71** | **Delirium** | Anticholinergics, Antipsychotics  Benzodiazepines, Sedative hypnotics* |
| **N87** | **Parkinson disease** | Antipsychotics, Metoclopramide |
| **P70** | **Dementia/ Cognitive impairment** | Anticholinergics, Antipsychotics  Benzodiazepines, TCAs |
| **L72~L76** | **Falls** | Antipsychotics, Benzodiazepines  Opioids, Sedative hypnotics  TCAs, Thioridazine |
| **N07; N88** | **Epilepsy/Seizures** | Antipsychotics, Bupropion, Thioridazine |
| **P29** | **Behavioural and psychological symptoms of dementia** | Antipsychotics |
| **P73; P76** | **Depression** | Methyldopa |
| **T99** | **Hypokalaemia** | Thiazide diuretics |
| **K88** | **Postural hypotension** | Alpha-adrenoreceptor antagonists  Chlorpromazine, TCAs, Thioridazine |
| **T87** | **Diabetic hypoglycemia** | Beta blocking agents |
| **F93** | **Glaucoma** | Anticholinergics, TCAs |
| **D12** | **Chronic constipation** | Anticholinergics, CCBs, Methyldopa  Opioids, TCAs |
| **U99** | **Chronic kidney disease** | NSAIDs |
| **D85; D86** | **Peptic ulcer disease** | Aspirin, NSAIDs (Non-COX-2 selectvie agents) |
| **U02; U05; U07; U13; U29; Y06** | **Lower urinary tract symptoms** | Anticholinergics, Chlorpromazine, TCAs |
| **U08** | **Urinary retention** | Anticholinergics, Chlorpromazine, TCAs |
| **Y85** | **Benign prostatic hyperplasia** | Anticholinergics, TCAs |
| **U04** | **Urinary incontinence** | Alpha-adrenoreceptor antagonists in women, TCAs |
| **T92** | **Gout** | Thiazide diuretics |
| **L95** | **Osteoporosis** | Corticosteroids |
| **X76** | **Breast cancer** | Oestrogens |
| **R95** | **Chronic obstructive pulmonary disease** | Benzodiazepines, Beta blocking agents |
| **R96** | **Asthma** | Benzodiazepines, Beta blocking agents |
